# Efficacy of 5-FU Combined to Na[trans-RuCl_4_(DMSO)Im], A Novel Selective Antimetastatic Agent, on the Survival Time of Mice With P388 Leukemia, P388/DDP subline and MCa Mammary Carcinoma

**DOI:** 10.1155/MBD.1995.195

**Published:** 1995

**Authors:** M. Coluccia, G. Sava, G. Salerno, A. Bergamo, S. Pacor, G. Mestroni, E. Alessio

**Affiliations:** 1 Department of General Pathology and Oncology University of Bari Italy; 2 Institute of Pharmacology and Pharmacognosy University of Trieste Trieste Italy; 3 Department of Chemical Sciences University of Trieste Trieste Italy; 4 Fondazione Callerio Institutes of Biological Research Via A. Fleming, 22 - 31 Trieste 1 - 34127 Italy

## Abstract

The combinational treatment between the selective antimetastatic agent, sodium-*trans*-rutheniumtetrachloridedimethylsulfoxideimidazole, Na[*trans*-RuCl_4_(DMSO)Im], and the cytotoxic drug 5-fluorouracil (5-FU) on primary tumor growth and on the survival time of experimental tumors results in an effect significantly greater than that of each single agent used alone either with the solid metastasizing MCa mammary carcinoma of the CBA mouse or with the lymphocytic leukemia P388 and its platinum resistant P388/DDP subline. Thus the inorganic compound Na[*trans*-RuCl_4_(DMSO)Im], known for its potent and selective antimetastatic effects, positively interacts with the antitumor action of an organic anticancer agent such as 5-FU on both a solid metastasizing tumor and a tumor of lymphoproliferative type. In particular, the effects of the combinational treatment on the survival time of tumor bearing mice seem to be related to the selective antimetastatic activity of the ruthenium complex that joins the potent cytotoxicity of 5-FU for the tumor. Moreover, these data show that Na[*trans*-RuCl_4_(DMSO)Im] is almost as effective on the subline of P388 made resistant to cisplatin as it was on the parental line.


### EFFICACY OF 5-FU COMBINED TO Na[trans-$RuCl_4$(DMSO)Im], A NOVEL SELECTIVE ANTIMETASTATIC AGENT, ON THE SURVIVAL TIME OF MICE WITH P388 LEUKEMIA, P388/DDP SUBLINE AND MCa MAMMARY CARCINOMA

M. Coluccia[1], G. Sava[2*], G. Salerno[4], A. Bergamo[4], S. Pacor[1], G. Mestroni[3], and E. Alessio[3]

[1] Department of General Pathology and Oncology, University of Bari
[2] Institute of Pharmacology and Pharmacognosy, University of Trieste
[3] Department of Chemical Sciences, University of Trieste
[4] Fondazione Callerio, Institutes of Biological Research,
Via A. Fleming, 22 - 31, I - 34127 Trieste, Italy

### ABSTRACT

The combinational treatment between the selective antimetastatic agent, sodium-*trans*-rutheniumtetrachloridedimethylsulfoxideimidazole, Na[*trans*-$RuCl_4$(DMSO)Im], and the cytotoxic drug 5-fluorouracil (5-FU) on primary tumor growth and on the survival time of experimental tumors results in an effect significantly greater than that of each single agent used alone either with the solid metastasizing MCa mammary carcinoma of the CBA mouse or with the lymphocytic leukemia P388 and its platinum resistant P388/DDP subline. Thus the inorganic compound Na[*trans*-$RuCl_4$(DMSO)Im], known for its potent and selective antimetastatic effects, positively interacts with the antitumor action of an organic anticancer agent such as 5-FU on both a solid metastasizing tumor and a tumor of lymphoproliferative type. In particular, the effects of the combinational treatment on the survival time of tumor bearing mice seem to be related to the selective antimetastatic activity of the ruthenium complex that joins the potent cytotoxicity of 5-FU for the tumor. Moreover, these data show that Na[*trans*-$RuCl_4$(DMSO)Im] is almost as effective on the subline of P388 made resistant to cisplatin as it was on the parental line.

### INTRODUCTION

On the wave of enthusiasm for the positive results obtained with platinum complexes[1], the search for novel anticancer agents has brought to the characterization of new compounds based on complexes of transition metals. Of these, ruthenium complexes attracted the interest of many groups because of some promising chemical characteristic and pharmacologic activity in different models of experimental tumors[2-4]. One of the last compounds that have been discovered is certainly Na[*trans*-$RuCl_4$(DMSO)Im], a drug of the future endowed with a particular effectiveness against solid tumor metastases[5,6].

Considering that anticancer chemotherapy is practically always done by drugs active on primitive tumors, we thought it worthwhile testing the effects of a combination with the selective antimetastatic agent Na[*trans*-$RuCl_4$(DMSO)Im] and a cytotoxic drug of a large use such as 5-fluorouracil (5-FU). The purpose of the study was that of measuring the existence of a combination of effects on the primary and on the metastatic tumor, with a significant amelioration of life-time expectancy. The study was made either on a solid metastasizing tumor, the MCa mammary carcinoma of the CBA mouse[7] or with a tumor of lymphoproliferative type, the P388 lymphocytic leukemia and its platinum resistant P388/DDP subline.

### MATERIALS AND METHODS

Compounds. 5-FU was kindly supplied by the Drug Synthesis and Chemistry Branch, NCI, NIH, Bethesda, MD, USA, and Na[*trans*-$RuCl_4$(DMSO)Im] was prepared according to already reported conditions. Both drugs were administered parentherally in volumes of 0.1 ml/10gr body weight of isotonic sterile saline.

Animals. CBA female mice of a locally established breeding colony, originally obtained from Chester Beatty Institute of London, UK, were used together with BD2F1 female mice purchased from Charles River, Calco, Como , Italy. All animals were of 22±1 gr body weight and bout 5 to 6 weeks old. Animal studies were carried out according to the guidelines currently in force in Italy (DL 116, 27/1/1992) and in compliance to the 'Guide for the care and use of laboratory animals' DHHS Publ. No (NIH)86-23. Bethesda, MD: NIH, 1985.

Tumors. MCa mammary carcinoma was transplanted i.m. into the calf of the left hind leg of CBA mice ($10^6$ viable tumor cells per mouse). The single cell preparation was performed according to standard procedures, starting from tumor masses obtained by donors with 2-week old tumors[6].

### RESULTS

Effects on MCa mammary carcinoma. In female CBA mice Na[*trans*-RuCl4(DMSO)Im] was given i.v. at 60 mg/Kg/day on days 1,5,9,13 from i.m. implantation of $10^6$ cells of MCa mammary carcinoma; treatment with 5-FU was made i.p. on the same days and at two dose levels of 30 and 50 mg/kg/day. The dose used for Na[*trans*-RuCl4(DMSO)Im] is one of those already shown active on survival time and on metastasis formation in mice with solid metastasizing tumors including MCa mammary carcinoma[8], whereas those chosen for 5-FU represent the doses that resulted more effective in preliminary studies of tumor inhibition.

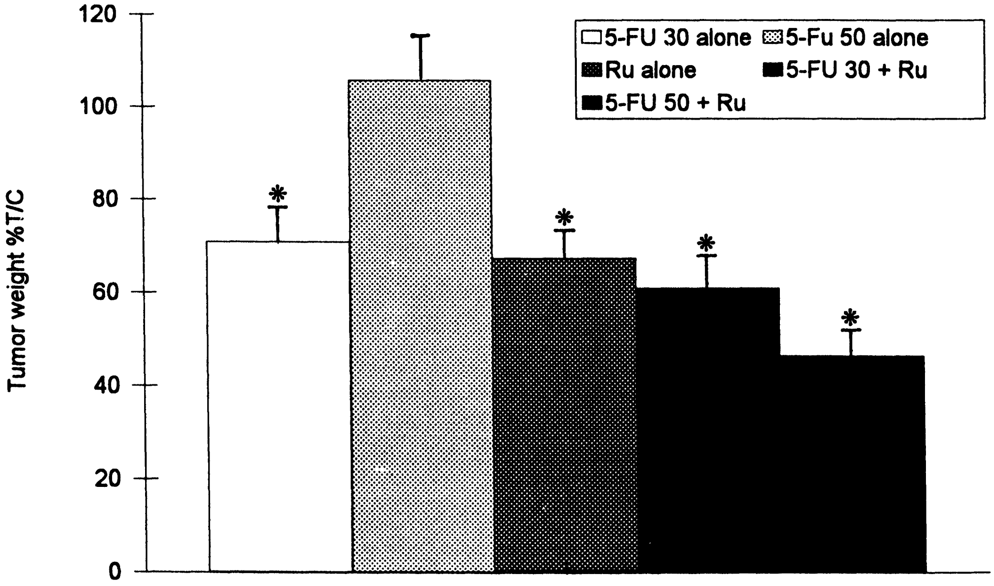

**Figure 1.** Effects of Na[*trans*-RuCl$_4$(DMSO)Im] and of 5-FU used alone or in combination on primary tumor growth in mice bearing MCa mammary carcinoma.

Groups of 10 CBA female mice, implanted i.m. with MCa mammary carcinoma on day 0, were given i.p. 5-FU and i.v. Na[*trans*-RuCl$_4$(DMSO)Im] on days 1,5,9,13. *: mean significantly different from untreated controls, Student-Newman-Keuls test, $p<0.05$.

On primary tumor (Figure 1), a statistically significant reduction was caused by 5-FU at 50 mg/kg/day and by Na[*trans*-RuCl$_4$(DMSO)Im], as determined on day 16 from tumor implantation (i.e. 72 hr after last day of treatment); the combination of the two drugs was more effective than each single compound if 5-FU were given at 50 mg/kg/day ($p<0.05$ *vs* 5-FU alone and *vs* Na[*trans*-RuCl$_4$(DMSO)Im] alone).

The i.p. treatment with 5-FU, at the dose of 30 mg/kg/day, and the i.v. administration of 60 mg/kg/day Na[*trans*-$RuCl_4$(DMSO)Im] caused a statistically significant prolongation of the survival time of the treated mice *vs* controls ($p<0.05$) (the statistical analysis applied is the Kaplan-Meyer test for survivals). The combinational treatment with the two compounds caused an even greater effect ($p<0.01$ *vs* controls and $p<0.05$ *vs* 5-FU alone and Na[*trans*-$RuCl_4$(DMSO)Im] alone). The combined treatment between 5-FU, at 50 mg/kg/day, and 60 mg/kg/day Na[*trans*-$RuCl_4$(DMSO)Im] caused a statistically significant prolongation of the life-span ($p<0.05$) not different from that observed with each drug used as single agent.

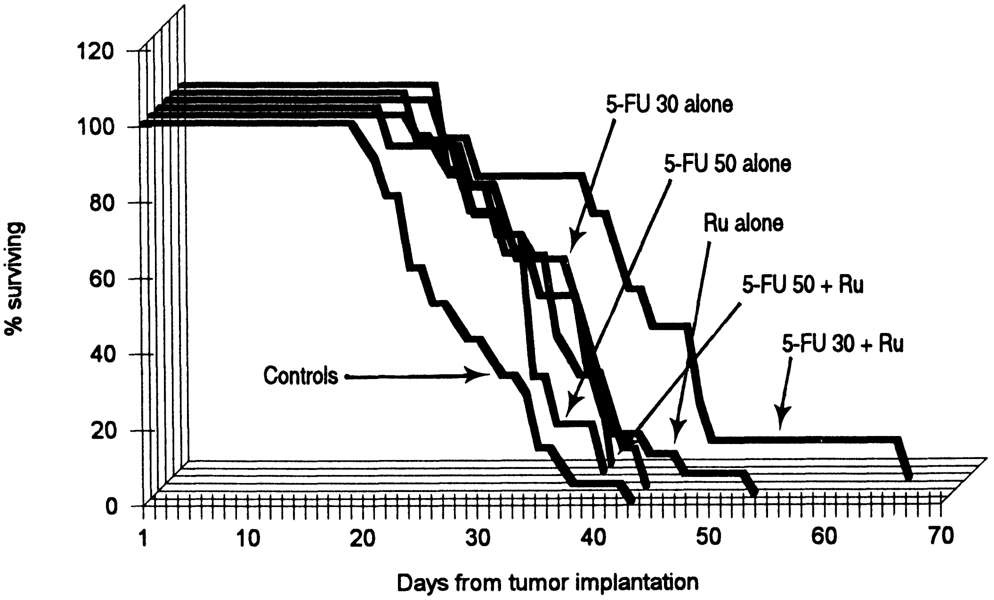

**Figure 2.** Effects of Na[*trans*-$RuCl_4$(DMSO)Im] and of 5-FU used alone or in combination on the survival time of mice bearing MCa mammary carcinoma.

Details on the experimental procedure are reported in Figure 1.

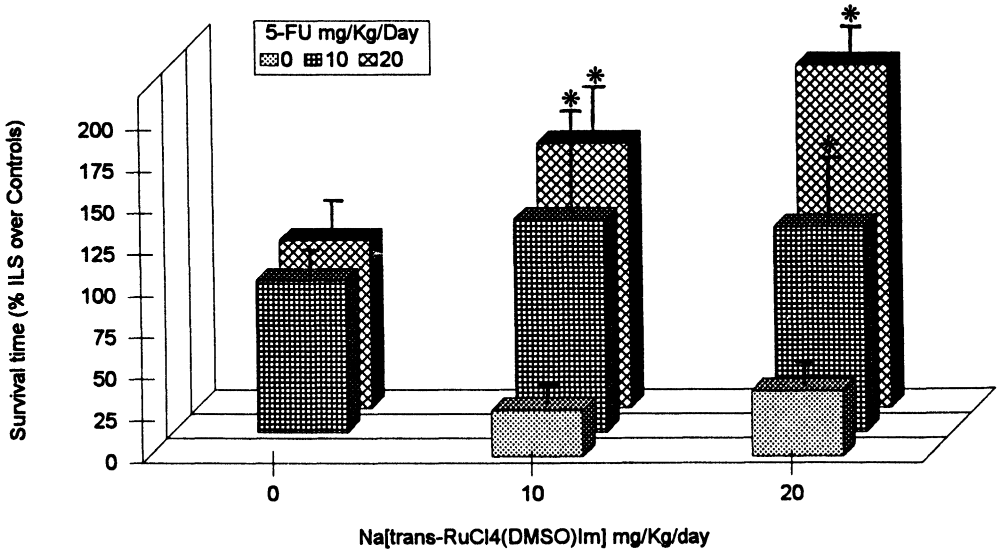

**Figure 3.** Effects of Na[*trans*-$RuCl_4$(DMSO)Im] and of 5-FU used alone or in combination on the survival time of mice bearing P388 leukemia.

Groups of 5 BD2F1 female mice, implanted i.p. with $10^6$ leukemic cells of P388 on day 0, were given the test compounds simultaneously i.p. on days 1-7. All treated groups statistically differ from controls; *: $p<0.05$ *vs* each single treatment, test of Student-Newmann-Keuls.

Effects on P388 leukemia. The effects of *in vivo* treatment with 5-FU alone (treatment with 10 or 20 mg/kg/day) on P388 leukemia and on its cisplatin resistant subline was generally more effective than that with Na[*trans*-RuCl$_4$(DMSO)Im] at the same dosages and treatment schedule (Figure 3 and Figure 4) ($p<0.05$).

The combined and simultaneous treatment with 5-FU and Na[*trans*-RuCl$_4$(DMSO)Im] showed a prolongation of the life span of the treated animals greater than that observed with each single agent ($p<0.05$) and in some cases greater than the sum of the singular effects of each single agent (20 mg/kg/day 5-FU plus 20 mg/kg/day Na[*trans*-RuCl$_4$(DMSO)Im] on the P388 parental line).

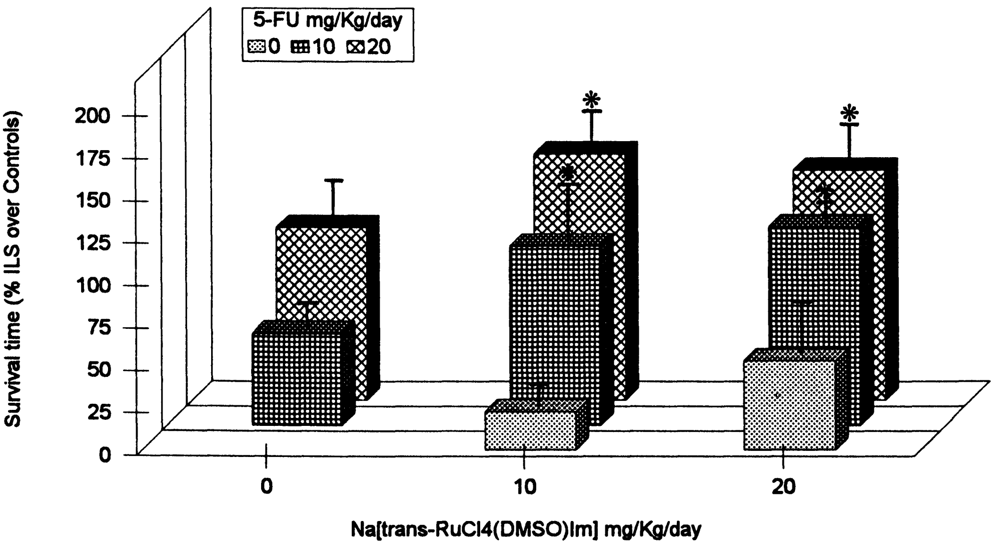

**Figure 4.** Effects of Na[*trans*-RuCl$_4$(DMSO)Im] and of 5-FU used alone or in combination on the survival time of mice bearing P388/DDP leukemia subline.

The experimental condition used was identical to that reported in Figure 3.

### DISCUSSION

These data show that the inorganic compound Na[*trans*-RuCl$_4$(DMSO)Im] can combine its antitumor effects to those of an organic anticancer agent such as 5-FU on both a solid metastasizing tumor and a tumor of lymphoproliferative type. The choice of 5-FU for this test was based on the proposed role of ruthenium(III) complexes for treating colo-rectal tumors[2,9] where 5-FU is the drug most often employed. Anyhow, it must also be emphasized that the aim of this paper was simply that of giving evidence that the emerging antimetastatic drug Na[*trans*-RuCl$_4$(DMSO)Im], in combinational treatments, does not reduce the antitumor action of a drug of common clinical use and that with some combinations it might also increase the overall response of the tumor being treated. In fact, it is interesting to note that, when the effect of the combined treatment is lower that that expected from the sum of the effects of each individual drug, on the solid metastasizing tumor it is at least as great as that of Na[*trans*-RuCl$_4$(DMSO)Im] and on the P388 tumors it is equivalent to that of 5-FU; in both cases with no modification of host toxicity, expressed as % loss of body weight gain *vs* untreated controls. Thus Na[*trans*-RuCl$_4$(DMSO)Im] has no negative effects on the antitumor activity of 5-FU and to some extent, it potentiates the effects of 5-FU.

The effects of the combinational treatment on the survival time of mice with MCa mammary carcinoma should be attributed to the antimetastatic activity of the ruthenium complex. The reduction of metastases by the ruthenium complex, besides the selective conditions in which it has often been tested[8,10], is thus therapeutically useful even in the present experimental model, a paradigm that does not emphasize the role of distant metastases for survival in that the presence contributed to the overall antimetastatic activity should be rouled out since in separate experiments this drug was completely ineffective on the formation of spontaneous lung metasases in mice with i.m. implants of MCa mammary carcinoma. The effect of Na[*trans*-$RuCl_4$(DMSO)Im] on tumor metastases might explain the greater efficacy of the combinational treatment *vs* single agents also on P388 tumors. In fact, ruthenium complexes with dimethylsulfoxides have been shown to inhibit also the formation of brain metastases of leukemic tumors[10,11]

In addition, the studies with P388 tumors point out the efficacy of Na[*trans*-$RuCl_4$(DMSO)Im] on the subline of P388 made resistant by cisplatin; an activity close to that shown on the parental line. This effect stresses once more that Na[*trans*-$RuCl_4$(DMSO)Im], still belonging to the so called platinum group, does not share with cisplatin the mechanims of antitumor activity nor the susceptibility of the tumor lines. Thus, the two drugs belong to two different classes of anticancer agents as already evidenced in detail on solid metastasizing tumors[8], indicating that this type of ruthenium compouds should not be considered as substitutive of cisplatin or of other platinum analogs.

**ACKNOWLEDGEMENTS**: This work was done with contributions by MURST 60%, by CNR ACRO and by Cassa di Risparmio di Trieste-Fondazione.

### REFERENCES

1. M.J. Cleare and P.C. Hydes. In: (Marcel Dekker Ed.), *Metal Ions in Biological Systems*, **vol 11**, New York, 1979, pp. 1-62.
2. B.K. Keppler, M. Henn, U.M. Juhal, M.R. Berger, R. Niebel and F.E. Wagner. In: (M.J. Clarke Ed.), *Progress in Clinical Biochemistry and Medicine - Ruthenium and other non-platinum metal complexes in cancer chemotherapy*, Springer-Verlag, Heidelberg, 1989, pp. 41-69.
3. G. Sava, S. Pacor, F. Bregant, V. Ceschia and G. Mestroni. *Anti Cancer Drugs*, **1**: 99-108, 1990.
4. M.J. Clarke. In: (M.J. Clarke Ed.), *Progress in Clinical Biochemistry and Medicine - Ruthenium and other non-platinum Metal Complexes in Cancer Chemotherapy*, Springer-Verlag, Heidelberg, 1989, pp. 25-39.
5. G. Sava, S. Pacor, G. Mestroni and E. Alessio. *Clin Expl Metastasis* **10**: 273-280, 1992.
6. R. Gagliardi, G. Sava, S. Pacor, G. Mestroni and E. Alessio. *Clin Expl Metastasis* **12**: 93-100, 1994.
7. M. Poliak-Blazi, M. Boranic, B. Marzan and M. Radacic. *Vet Arh* **51**: 99-107, 1981.
8. G. Sava, S. Pacor, M. Coluccia, M. Mariggiò, M. Cocchietto, E. Alessio, G. Mestroni. *Drug Investigation* **8**: 150-161, 1994.
9. F.T. Garzon, M.R. Berger, B.K. Keppler, D. Schmahl. *Cancer Chemother Pharmacol* **19**: 347-349, 1987.
10. G. Sava, S. Pacor, A. Bergamo, M. Cocchietto, G. Mestroni, E. Alessio. *Chem Biol Interactions* in press 1995.
11. M. Coluccia, G. Sava, F. Loseto, A. Nassi, A. Boccarelli, D. Giordano, E. Alessio, G. Mestroni. *Eur J Cancer* **29A**: 1873-1879, 1993.

**Received: February 14, 1995 - Accepted: March 1, 1995 - Received camera-ready revised version: April 19, 1995**

